# Transcriptomic recurrence score improves recurrence prediction for surgically treated patients with intermediate‐risk clear cell kidney cancer

**DOI:** 10.1002/cam4.5399

**Published:** 2022-11-17

**Authors:** Neal Patel, Alexander Hakansson, Shinji Ohtake, Peter Muraki, James A. Proudfout, Yang Liu, Lisa Webber, Arkaitz Ibarra, Vinnie Y. T. Liu, Elai Davicioni, Karim Chamie, Allan Pantuck, Brian Shuch

**Affiliations:** ^1^ Department of Urology Institute of Urologic Oncology, University of California, Los Angeles Los Angeles California USA; ^2^ Veracyte, Inc South San Francisco California USA

**Keywords:** adjuvant therapy, kidney cancer, transcriptomics

## Abstract

**Background:**

Risk stratification of kidney cancer patients after nephrectomy may tailor surveillance intensity and selection for adjuvant therapy. Transcriptomic approaches are effective in predicting recurrence, but whether they add value to clinicopathologic models remains unclear.

**Methods:**

Data from patients with clear cell renal cell carcinoma (ccRCC) was downloaded from The Cancer Genome Atlas. Clinicopathologic variables were used to calculate SSIGN (stage, size, grade, and necrosis) scores. The 16 gene recurrence score (RS) signature was generated using RNA‐seq data. Transcriptomic risk groups were calculated using the original thresholds. SSIGN groups were divided into low, intermediate, and high risk. Disease‐free status was the primary endpoint assessed.

**Results:**

SSIGN and RS were calculated for 428 patients with non‐metastatic ccRCC. SSIGN low‐, intermediate‐, and high‐risk groups demonstrated 2.7%, 15.2%, and 27.5%, 3‐year recurrence risk, respectively. On multivariable analysis, the RS was associated with disease‐free status (sub‐distribution hazard ratio (sHR) 1.43 per 25 RS [95% CI (1.00–1.43)], *p* = 0.05). By risk groups, RS further risk stratified the SSIGN intermediate‐risk group (sHR 2.22 [95% CI 1.10–4.50], *p* = 0.03). SSIGN intermediate‐risk patients with low and high RS had a 3‐year recurrence rate of 8.0% and 25.2%, respectively. Within this risk group, the area under the curve (AUC) at 3 years was 0.69 for SSIGN, 0.74 for RS, and 0.78 for their combination.

**Conclusions:**

Transcriptomic recurrence scores improve risk prediction even when controlling for clinicopathologic factors. Utility may be best suited for intermediate‐risk patients who have heterogeneous outcomes and further refinement for clinical utility is warranted.

## 1 INTRODUCTION

Patients undergoing radical nephrectomy with curative intent for localized renal cell carcinoma who are found to have high‐risk disease have a significant risk of developing distant metastases after surgery.^1^ Despite recurrence rates of up to 50% for the highest risk patients, adjuvant therapy has not been widely utilized due to cumulative toxicity and conflicting evidence on the benefit of tyrosine kinase inhibitors' (TKIs), including a lack of overall survival benefit.^2^, ^3^ Recently, however, adjuvant pembrolizumab was shown to significantly improve disease‐free survival.^4^ Given the risk of long‐term toxicity, significant costs, and the significant number of patients cured with nephrectomy alone, identifying the subset of patients who are destined to recur would be beneficial to avoid overtreatment.

Current risk stratification tools largely designed from retrospective, single institution data rely on clinical and pathological characteristics to determine prognosis and are imperfect at predicting recurrences. Although multiple nomograms such as the Memorial Sloan Kettering Cancer Center nomogram, Stage, Size, Grade and Necrosis score (SSIGN) and the University of California Integrated Staging System (UISS)^,^ have been developed, their performance in detecting recurrences decreases substantially when applied to clinical trial data of largely high‐risk patients.^5^, ^6^, ^7^, ^8^, ^9^ While these tools may improve upon stage and grade alone, they are not recommended to further define risk and select individuals for additional treatment by national guidelines.^10^

Genomic biomarkers have the potential to improve risk stratification as well as further refine treatment selection and are already used across several cancer types such as breast and prostate to help inform routine clinical management such as administering adjuvant therapy.^11^, ^12^, ^13^ Given that patients with high‐risk localized clear cell renal cell carcinoma recur frequently and cure thereafter is not readily achievable, there is an unmet clinical need to improve risk stratification in an era of new treatment options. Previously, a transcriptomic signature was developed from a novel 16 gene assay that was shown to be significantly associated with the risk of developing metastases in institution cohorts but had only modest improvement compared with clinicopathologic variables.^14^ In this study, we sought to externally validate the transcriptomic signature in a more heterogeneous cohort from The Cancer Genome Atlas (TCGA) and to evaluate its utility when combined with standard clinicopathologic risk groups.

## 2 METHODS

### 2.1 Data source and patient cohort

The TCGA Pan RCC data was downloaded from cbioportal for Cancer Genomics (http://www.cbioportal.org/) and the Genomic Data Commons bioportal (https://portal.gdc.cancer.gov/). The dataset contains outcome data linked to detailed clinical information, somatic mutations, and mRNA expression counts as previously described. Eligible patients for analysis included those with (1) clear cell renal cell carcinoma (2) pathological stage I‐III and (3) complete clinicopathologic data. Patients were excluded from the analysis if they were (1) pathological stage IV, (2) metastatic or (3) non‐clear cell renal cell carcinoma. The Institutional Review Board deemed the study exempt because the study did not constitute human participant research and used a publicly available dataset.

### 2.2 Integrated staging system and recurrence score

Demographic and clinicopathologic variables were abstracted for eligible patients. Clinicopathologic variables were used to calculate the integrated staging system, SSIGN (stage, size, grade, and necrosis).^8^ SSIGN was used as the clinicopathologic staging system due to the high performance and availability of the data while other factors (such as performance status) were not available for UISS. SSIGN was divided into three risk groups: low (0–1), intermediate,^2^, ^3^, ^4^ and high (≥5). Recurrence scores (RS) were calculated based on the expression of 16 genes (11 cancer‐related genes associated with recurrence free survival and 5 housekeeping genes) as previously described by Rini et al.^14^ Recurrence scores are scaled from 0 to 100 and were subsequently stratified into three risk groups using the specified thresholds (low: RS <32, intermediate: RS 32–44, high: RS ≥45).^14^ Due to the reduced sample size within SSIGN strata, the intermediate‐ and high‐risk RS were combined for some analysis.

### 2.3 Statistical methods

Continuous and categorical clinical and pathological variables were reported as medians (interquartile ranges, IQR) and counts (proportions), respectively. Comparisons of clinical and pathological variables between RS risk groups were performed using the Kruskal–Wallis test for continuous variables and chi‐squared tests for categorical variables. Fine and Gray models were used to determine the associations between time to recurrence and clinical and pathological characteristics, RS, and SSIGN, with death before recurrence treated as a competing risk.^15^ A multivariable Fine and Gray model was also fit with RS and SSIGN concurrently. Sub‐distribution hazard ratios (sHR) and their 95% confidence intervals were derived from these models. Cumulative incidence estimates were reported for SSIGN and RS. Area under the receiver operating characteristic (ROC) curves at 3 years were also generated. All statistical tests were two sided with p‐values ≤0.05 indicating statistical significance. All analyses were performed using R version 4.0.3.

## 3 RESULTS

Baseline clinical and pathological characteristics of the 428 patients with non‐metastatic ccRCC are presented in Table 1, stratified by RS risk group. Higher RS group was associated with more aggressive clinicopathologic features. Tumor size increased with RS risk classification (6.5 cm (IQR 5.5–10.3) for RS high, 5.7 cm (IQR 4.0–8.5) for RS intermediate, and 4.5 cm (IQR 3.5–6.3) for RS low‐risk patients, *p* < 0.001). Patients within the RS high group had a higher frequency of Furman grade 4 disease (26.7% for RS high, 8.7% for RS intermediate, and 4.2% for RS low patients, *p* < 0.001) and lymph node‐positive disease (6.7% for RS high, 2.9% for RS intermediate, and 1.1% for RS low, *p* = 0.007). Patients classified RS high were also more likely to be classified as high SSIGN (41.7% for RS high, 31.1% for RS intermediate, and 11.3% for RS low patients, *p* < 0.001).

**TABLE 1:** Baseline characteristics of non‐metastatic clear cell patients who underwent surgical therapy in TCGA stratified by stage

	Stage I (*n* = 221, 60.05%)	Stage II (*n* = 47, 12.77%)	Stage III (*n* = 97, 26.36%)	*p*‐value
Age (years), median (IQR)	59 [50–68]	58 [48–68]	63 [52–71]	0.034*
Sex				0.124
Female	87 (39.37)	11 (23.40)	29 (29.90)	
Male	134 (60.63)	36 (76.60)	68 (70.10)	
Primary tumor size, median (IQR)	4.00 [3.10–5.20]	9.00 [7.80–10.50]	7.10 [5.50–10.57]	<0.001*
Tumor necrosis				0.85
Absent	85 (91.40)	12 (85.71)	31 (88.57)	
Present	8 (8.60)	2 (14.29)	4 (11.43)	
Tumor grade				<0.001*
G1	12 (5.45)	1 (2.17)	0 (0.00)	
G2	131 (59.55)	20 (43.48)	31 (31.96)	
G3	71 (32.27)	20 (43.48)	50 (51.55)	
G4	5 (2.27)	4 (8.70)	16 (16.49)	
GX	1 (0.45)	1 (2.17)	0 (0.00)	
T stage				<0.001*
T1	220 (99.55)	0 (0.00)	2 (2.06)	
T2	0 (0.00)	47 (100.00)	1 (1.03)	
T3	1 (0.45)	0 (0.00)	94 (96.91)	
N stage				<0.001*
N0	82 (37.10)	31 (65.96)	49 (50.52)	
N1	0 (0.00)	0 (0.00)	10 (10.31)	
NX	139 (62.90)	16 (34.04)	38 (39.18)	
SSIGN				<0.001*
Low	155 (70.14)	7 (14.89)	0 (0.00)	
Intermediate	62 (28.05)	36 (76.60)	37 (38.14)	
High	4 (1.81)	4 (8.51)	60 (61.86)	
Recurrence score				<0.001*
Low	164 (74.21)	22 (46.81)	43 (44.33)	
Intermediate	35 (15.84)	15 (31.91)	36 (37.11)	
High	22 (9.95)	10 (21.28)	18 (18.56)	

* Statistical significance.

**FIGURE 1:**
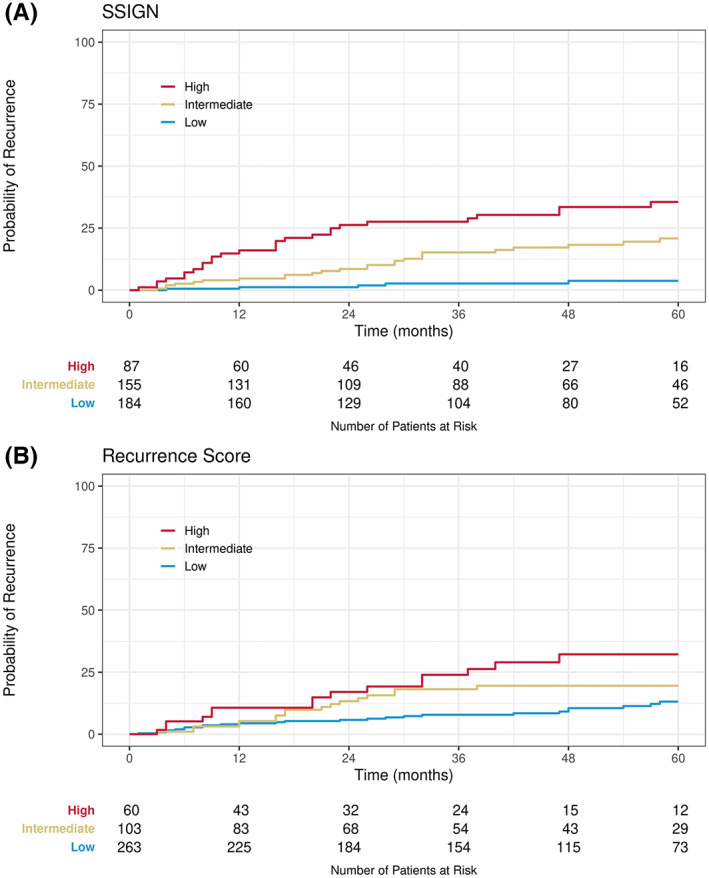
Cumulative incidence of recurrence by SSIGN risk groups (A) and RS risk groups (B).

Disease recurrence cumulative incidence plots were generated for RS and SSIGN. Median follow‐up time for non‐recurring patients was 43 months and overall recurrence rate was 16.8%. As expected, higher risk groups have greater recurrence risk (Figure 1). Three‐year recurrence rates were 7.8% [95% CI 4.3%–11.3%] for RS low, 18.2% [95% CI 10.0%–26.3%] for RS intermediate, and 23.9% [95% CI 0.11.8%–36.1%] for RS high. Three‐year recurrence rates were 2.7% [95% CI 0.6%–5.3%] for SSIGN low, 15.2% [95% CI 9.0%–21.4%] for SSIGN intermediate, and 27.5% [95% CI 17.6%–37.5%] for SSIGN high.

When analyzed as continuous variables, both higher SSIGN score (sHR 1.39, [95% CI 1.26–1.54], *p* < 0.001) and RS (sHR 2.04 per 25 RS, [95% CI 1.49–2.79], *p* < 0.001) were associated with increased rates of disease recurrence (Table 2). When treated as categorical risk groups, compared with the RS low‐risk group, only RS high was associated with an increase in recurrence rate (sHR 2.95 vs. RS low, [95% CI 1.70–5.12], *p* < 0.001). Compared with the SSIGN low‐risk group, both SSIGN intermediate‐ and high‐risk groups were associated with an increase in recurrence rates (sHR 4.11, [95% CI 1.99–8.49], *p* < 0.001 and sHR 8.16, [95% CI 3.92–16.98], *p* < 0.001, respectively). Tumor stage, tumor size, and lymph node involvement were also significantly associated with recurrence (Table 2).

**TABLE 2:** Univariable Fine‐Gray model results for recurrence with clinical and pathological variables

	Subdistribution Hazard ratio (95% CI)	*p*‐value
Age (per year)	1.00 (0.98–1.02)	0.72
Tumor size (per cm)	1.21 (1.14–1.28)	<0.001
Stage		
T2	2.25 (1.10–4.61)	0.03
T3	3.72 (2.23–6.20)	<0.001
Furman grade		
G2	1.01 (0.15–6.79)	0.99
G3	1.82 (0.27–12.14)	0.54
G4	4.14 (0.59–28.81)	0.15
Necrosis	1.99 (0.57–6.93)	0.28
Lymph node positive	10.93 (4.77–25.06)	<0.001
Recurrence score (Per 25 RS)	2.04 (1.49–2.79)	<0.001
Recurrence score		
Intermediate	1.64 (0.94–2.86)	0.08
High	2.95 (1.70–5.12)	<0.001
SSIGN score	1.39 (1.26–1.54)	<0.001
Intermediate	4.11 (1.99–8.49)	<0.001
High	8.16 (3.92–16.98)	<0.001

**FIGURE 2:**
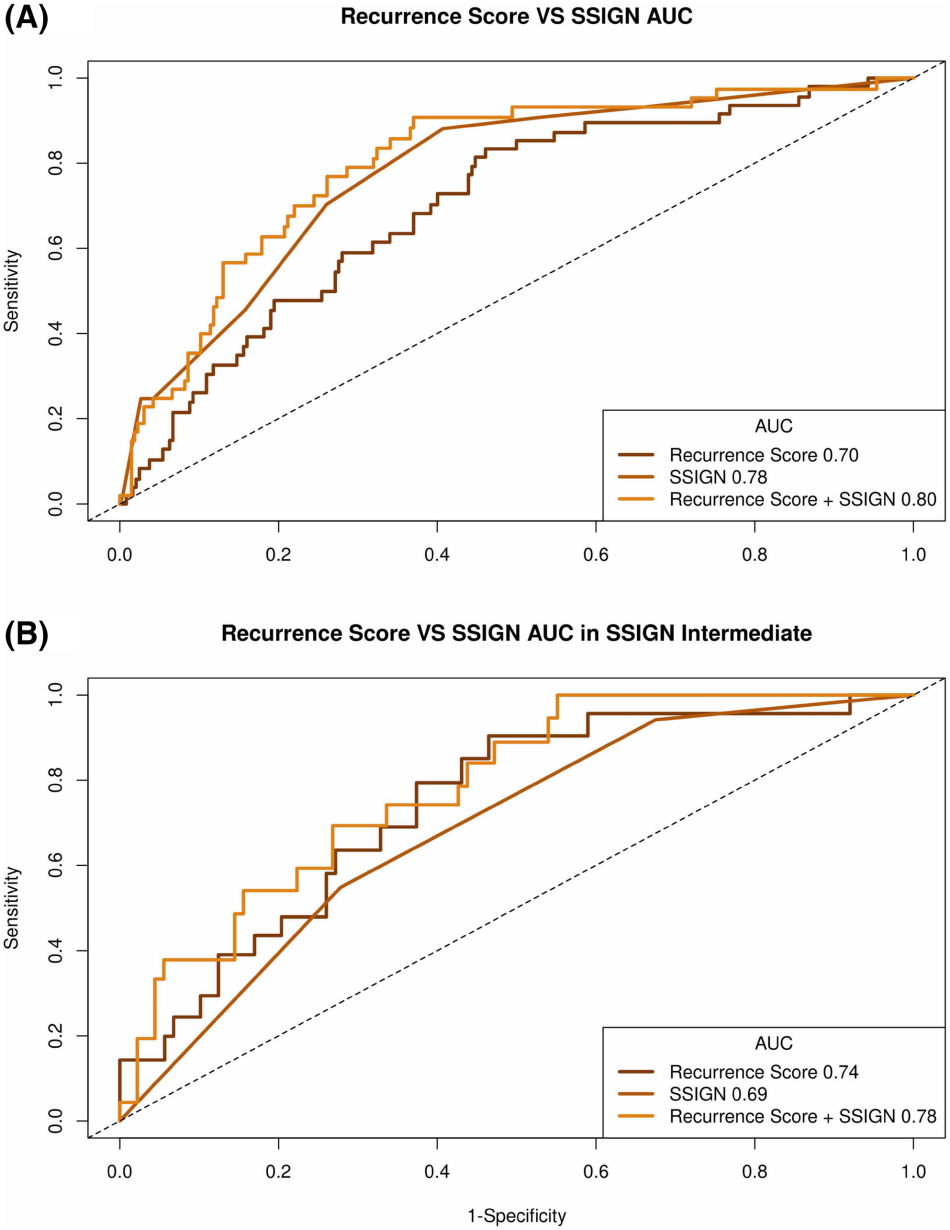
Area under the curve at three years for (A) RS, SSIGN and combination (B) within SSIGN intermediate risk group

In the multivariable analysis (MVA) model, both SSIGN score (sHR 1.35, [95% CI 1.21–1.50] *p* < 0.001) and RS (sHR 1.43 per 25 RS, [95% CI 1.00–2.04], *p* < 0.050) remained significantly associated with recurrence when analyzed as a continuous variable (Table 3). In the MVA of categorical SSIGN and RS risk groups, both the SSIGN intermediate‐ and high‐risk groups remained significantly associated with recurrence (sHR 3.80 [95% CI 1.85–7.84], *p* < 0.*p* < 0.001, and sHR 7.04 [95% CI 3.28–15.10], *p* < 0.001, respectively), while only the high‐risk RS group was significantly associated with recurrence (sHR 1.84 vs. low, [95% CI 1.03–3.26], *p* = 0.040). Discrimination for the recurrence endpoint was examined using ROC curve analysis for RS, SSIGN, and the combination of SSIGN plus RS. The combination of SSIGN and RS performed the best in this analysis with an area under the curve (AUC) of 0.80 followed by SSIGN alone with an AUC of 0.78 and RS with an AUC of 0.70 (Figure 2A). Within the SSIGN intermediate risk, RS had an AUC of 0.74 (Figure 2B).

**TABLE 3:** Multivariable Fine‐Gray model results for recurrence with continuous and categorical recurrence score and SSIGN

		Subdistribution Hazard ratio (95% CI)	*p*‐value
Recurrence score	Continuous (Per 25 RS)	1.43 (1.00–2.04)	0.05*
	Intermediate vs. Low	1.15 (0.63–2.10)	0.65
	High vs. Low	1.84 (1.03–3.26)	0.04*
SSIGN score	Continuous	1.35 (1.21–1.50)	<0.001*
	Intermediate	3.80 (1.85–7.84)	<0.001*
	High	7.04 (3.28–15.10)	<0.001*

* Statistical significance.

**FIGURE 3:**
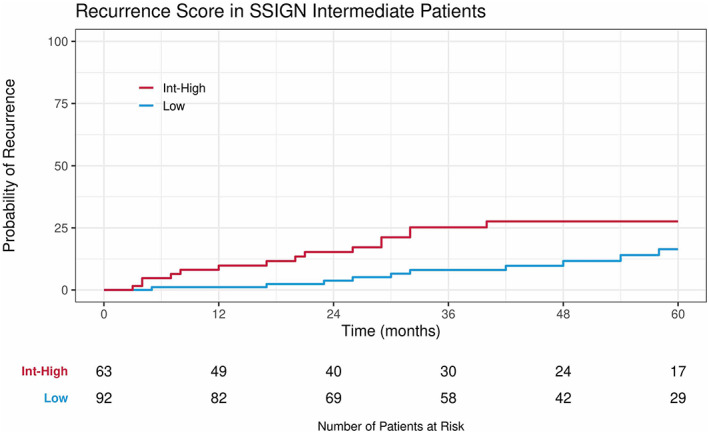
Cumulative incidence of recurrence by RS risk groups in the intermediate‐risk SSIGN subset.

As patients can be readily risk stratified by SSIGN grouping in the clinic, we investigated how RS varied within each SSIGN groups. RS was not significantly associated with recurrence in low‐ or high‐risk SSIGN patients (Table S1). Within the SSIGN intermediate group, three‐year recurrence rates were 8.0% [95% CI 1.8%–14.2%] in RS low and 25.2 [95% CI 13.5%–36.9%] in RS intermediate‐high (Figure 3). Continuous RS (sHR 2.99 per 25 RS [95% CI 1.48–6.02], *p* = 0.002) and categorical RS (intermediate‐high vs low RS sHR 2.22 [95% CI 1.10–4.50], *p* = 0.030) were both significantly associated with recurrence in SSIGN intermediate patients. The ROC curves for RS combined with SSIGN, SSIGN and RS had AUCs of 0.78, 0.69, and 0.74 respectively in this risk group (Figure S1). For intermediate‐risk SSIGN patients meeting key eligibility requirements for adjuvant treatment based on Keynote‐564, the recurrence rate was 33.1% [95% CI 10.4–55.9%] in the RS int‐high subset and 6.3% [95% CI 0.0%–18.5%] in the RS low subset (Figure S1); however, this difference was not statistically significant on the multivariable model.

## 4 DISCUSSION

Surgical therapy alone for high‐risk kidney cancer cures many patients, but the chance of recurrence can cause significant anxiety as systemic therapy for recurrences generally is non‐curative, leaving few long‐term survivors.^16^ As a result, there has been a longstanding interest in utilizing adjuvant therapy for high‐risk kidney cancer to treat patient with presumed micro‐metastatic disease. Although tyrosine kinase inhibitors (TKI) demonstrate clear benefit in patients with metastatic disease, the benefit in the adjuvant setting is mixed. The phase 3 S‐TRAC trial demonstrated improvement in disease‐free survival in patients treated with 1 year of standard dose sunitinib compared with placebo, however this benefit was not observed in other similar trials using VEGF tyrosine kinase inhibitors, thus the use of sunitinib has not been fully endorsed in National Comprehensive Cancer Network (NCCN) guidelines, largely due to toxicity concerns and a lack overall survival improvement.^2^, ^17^ Immunotherapy therapy appears to be effective in the adjuvant therapy and is better tolerated with approximately 83% of patients completing adjuvant pembrolizumab treatment. While there are expected immune‐related adverse events, only 7.6% of individuals required high‐dose steroids and there have been no reported treatment‐related deaths.^4^ In light of these findings, there was consensus among the NCCN guideline committee that adjuvant therapy be should offered to patients as an option with high‐risk kidney cancer after nephrectomy, and this recommendation has been incorporated into the most recent guidelines.^10^

If adjuvant therapy is to be widely implemented, there is, however, a significant risk of overtreatment and a large number of patients would be subjected to the associated toxicity and high cost of pembrolizumab to prevent a single recurrence. This is especially true among the non‐metastatic and non‐sarcomatoid cohorts in KEYNOTE 564, which composed the majority of patients enrolled in the trial, and where the benefit of adjuvant therapy was less pronounced. To date, in attempt to better risk stratify which patients have a high chance of recurrence after surgery, numerous prognostic models have been developed based on retrospective single institution data but unfortunately their performance declines substantially when applied to prospective clinical trial data.^5^ Why this occurs is unclear but may largely be due to the more uniform cohort contained in the clinical trials data. Noninvasive biomarkers like circulating tumor DNA and DNA methylation profiling are promising in solid oncology but may have a more limited role due to the lack of shedding in renal cancers even in the metastatic setting.^18^, ^19^ Genomic classification of resected tumors, however, could potentially help improve treatment selection. While there are clear recurrent driver mutations and distinct evolutionary subtypes common to ccRCC, even among these individual groups the molecular transcriptomic profiles vary widely.^20^, ^21^ Leveraging these unique transcriptomes could possibly better select which patients are at high risk of recurrence and would benefit from adjuvant therapy.

Prior reports have aimed to investigate specific transcriptomic features of high‐risk nephrectomy specimens. Two previously developed RNA based signatures, one using a 31 gene cell cycle progression (CCP) score and the second using a 16 gene assay, were shown to be correlated with developing recurrences even when controlling for clinicopathologic characteristics. However, major limitations of the CCP cohort included low recurrence rates, inclusion of low‐grade patients, and non‐clear cell histology in the cohort. The second classifier utilized a 16 gene signature that was developed in a cohort from Cleveland Clinic and validated in patients from Hôpital Foch and Hôpital Necker Enfants Malades. A second validation study using an independent cohort was then performed using patients who were enrolled in the S‐TRAC clinical trial. In this study only stage III kidney cancer patients were included,^17^ which is a limitation given that a significant number of patients with stage II tumors who have adverse pathological features such as high grade, microvascular invasion, and necrosis can recur, some of which could have been candidate for adjuvant treatment.

In this current study, we validated the utility of the RS in a heterogeneous, non‐single center cohort from the TCGA. We found that RS was prognostic as a continuous variable, even when controlling for clinicopathologic variables. As expected, worse RS also correlated with adverse clinical characteristics. Although patients can be risk stratified by routine clinicopathologic variables using SSIGN and other classifiers, there are only modestly accurate and there is room for improvement with a genomic classifier. This is especially true among clinically intermediate‐risk patients who have very variable prognoses. In general, most low‐risk RS patients will not recur and most high‐risk RS patients have enough risk where they will be offered adjuvant therapy regardless of classifier. With a modest number of events, its often the intermediate‐risk group that has enough heterogeneity in biology and outcome where genomic classifiers may play a large role. Such is the case in prostate cancer, where intermediate‐risk cancer is frequently separated into favorable and unfavorable subsets leading to divergent therapeutic treatments.^22^ Clinically, discriminating between patients in the intermediate SSIGN groups is meaningful because the overall risk of recurrence is lower with a wide range in clinical outcomes. By utilizing the RS in this group, the risk of recurrence can be better assessed, which could ultimately help improve the selection of candidates for adjuvant therapy. Lastly, among high‐risk SSIGN patients, the recurrence rate was 27.5%. Given that the majority of even the highest risk patients may be cured with nephrectomy alone, further refinement of a transcriptomic signature is needed to improve risk prediction in these patients.

With significant advances in systemic therapy, contemporary management of high‐risk localized disease will likely involve using systemic therapy earlier in the disease course. Genomic information harbored from the primary tumor has the potential to help better predict recurrence and can thereby, improve outcomes of patients by allowing for earlier escalation of treatment. Similarly, there is growing interest in utilizing transcriptomic signatures to help select treatment in metastatic clear cell kidney cancer. Discerning tumors with high angiogenesis gene expression and high immune gene expression can potentially help clinician navigate which tumor mechanism to target with systemic therapy.^20^, ^23^ Similarly, transcriptomic signatures could be used to help develop targeted treatment strategies in both the neoadjuvant and adjuvant setting to improve upon outcomes. In the era of personalized medicine, we must advocate as a community to embrace these approaches.

Our study is not without limitations. The study is retrospective in design and therefore validation in prospectively enrolled well‐annotated cohorts is necessary. Although the TCGA includes patients from multi‐institutions, the data may not be representative of the general population,^24^ especially given the small sample sizes within subgroups. Additional validation in prospective clinical data with longer follow‐up and larger patient numbers is needed to better understand the performance of such a signature in routine clinical practice. For this study, we focused on only the 16 gene score, but other prognostic signatures that have also been described were not evaluated. Furthermore, the survival follow‐up of patients that did not recur was limited (median 43 months) and some may exhibit late recurrence impacting long‐term prognostic capability. Lastly, although an assessment of disease recurrence remains valuable for patient selection, but our study does not attempt to predict response to therapy. A predictive biomarker may better aid in selection of patients for specific therapeutic approaches; however, its utility may not be relevant to other treatment strategies, something to recognize with a rapidly evolving field.^20^

## 5 CONCLUSION

Transcriptomic recurrence scores can help improve risk stratification of patients with intermediate SSIGN clinical risk patients. These patients have similar risk of recurrence as clinically high‐risk patients. Transcriptomic signatures can be combined with clinical information to improve risk assessment and further evaluation of clinical implementation is warranted.

## AUTHOR CONTRIBUTIONS

The authors confirm contribution to the paper as follows: study conception and design: B. Shuch, E. Davicioni, J. Proudfout, A. Ibarra; data collection: N. Patel, A. Hakansson, P. Muraki, S. Ohtake; analysis and interpretation of results: B. Shuch, E. Davicioni, A. Hakansoon, K. Chamie, A. Pantuck, Y. Liu, V. Liu, N. Patel; draft manuscript preparation: all authors. All authors reviewed the results and approved the final version of the manuscript.

## FUNDING INFORMATION

Veracyte, Inc.

## CONFLICT OF INTEREST

A. Hakansson, J. Proudfout, Y. Liu, L. Webber, A. Ibrarra, V. Liu, E. Davicioni are employees of Decipher Biosciences, a subsidiary of Veracyte, Inc. B. Shuch receives consulting fees from Veracyte, Inc.

## ETHICAL APPROVAL STATEMENT

The Institutional Review Board deemed the study exempt because the study did not constitute human participant research and used a publicly available dataset (UCLA IRB#22‐000680).

## Supporting information


Figure S1

Table S1

Table S2

## Data Availability

The data used in this study are publicly available and can be downloaded from cbioportal (http://www.cbioportal.org/) and the Genomic Data Commons bioportal (https://portal.gdc.cancer.gov/).
