# Supplementary material for: Transcriptomic recurrence score improves recurrence prediction for surgically treated patients with intermediate‐risk clear cell kidney cancer
Source: Cancer Med. 2022 Nov 17;12(5):6437–44. doi: 10.1002/cam4.5399 (PMC10028022; doi:10.1002/cam4.5399)

**Figure S1:** Cumulative incidence of recurrence in SSIGN intermediate patients by (A) adjuvant therapy eligibility (B) recurrence score risk in adjuvant therapy eligible patients (C) recurrence score risk in adjuvant therapy ineligible patients.

A.

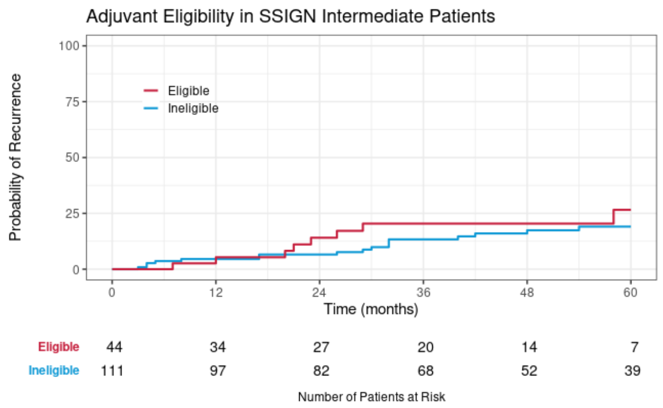


B.

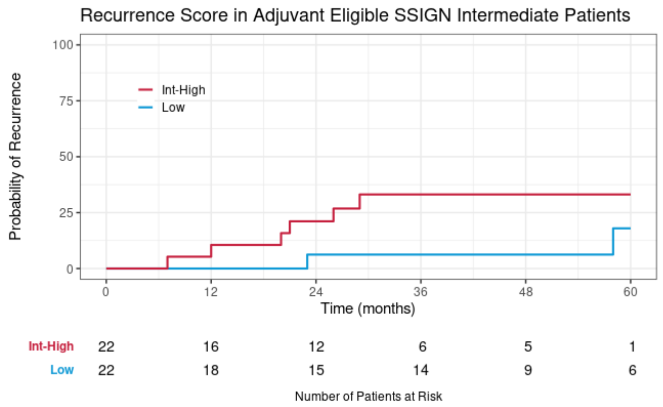


C.

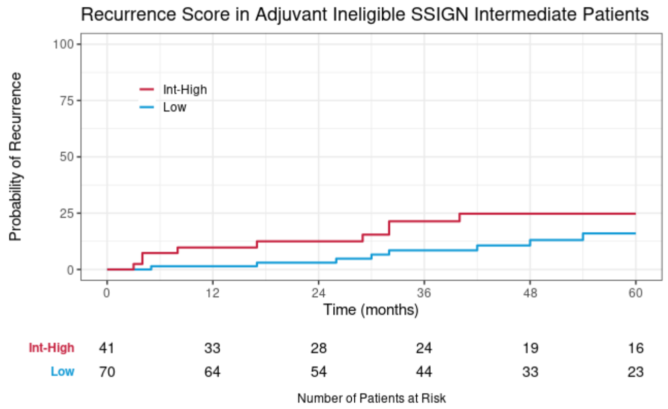

Supplement: Supplementary file 1 — Figure S1 [file CAM4-12-6437-s003.pdf]
