# Supplementary material for: Transcriptomic recurrence score improves recurrence prediction for surgically treated patients with intermediate‐risk clear cell kidney cancer
Source: Cancer Med. 2022 Nov 17;12(5):6437–44. doi: 10.1002/cam4.5399 (PMC10028022; doi:10.1002/cam4.5399)
Supplement: Supplementary file 2 — Table S1 [file CAM4-12-6437-s001.docx]

**Table S1:** Univariable (UVA) and multivariable (MVA) Fine and Gray model results for recurrence with SSIGN and recurrence score as covariates.

| SSIGN Low Risk Subset | | | |
| --- | --- | --- | --- |
| Model | Variable | Subdist. Hazard Ratio (95% CI) | p-value |
| UVA | Recurrence Score Int-High vs Low | 0.88 (0.19 - 4.07) | 0.87 |
|  | Recurrence Score (per 25) | 1.20 (0.53 - 2.71) | 0.66 |
|  | SSIGN Score | 1.41 (0.43 - 4.64) | 0.58 |
| MVA: Recurrence Score + SSIGN Score | Recurrence Score | 1.05 (0.44 - 2.53) | 0.91 |
|  | SSIGN Score | 1.37 (0.38 - 5.03) | 0.63 |
| SSIGN Intermediate Risk Subset | | | |
| Model | Variable | Subdist. Hazard Ratio (95% CI) | p-value |
| UVA | Recurrence Score Int-High vs Low | 2.22 (1.10 - 4.50) | 0.03 |
|  | Recurrence Score (per 25) | 2.99 (1.48 - 6.02) | 0.002 |
|  | SSIGN Score | 1.47 (0.94 - 2.28) | 0.09 |
| MVA: Recurrence Score + SSIGN Score | Recurrence Score | 2.82 (1.35 - 5.87) | 0.006 |
|  | SSIGN Score | 1.33 (0.83 - 2.14) | 0.23 |
| SSGIN High Risk Subset | | | |
| Model | Variable | Subdist. Hazard Ratio (95% CI) | p-value |
| UVA | Recurrence Score Int-High vs Low | 0.81 (0.40 - 1.66) | 0.57 |
|  | Recurrence Score (per 25) | 1.04 (0.64 - 1.72) | 0.86 |
|  | SSIGN Score | 1.24 (0.93 - 1.65) | 0.14 |
| MVA: Recurrence Score + SSIGN Score | Recurrence Score | 0.95 (0.56 - 1.61) | 0.85 |
|  | SSIGN Score | 1.25 (0.93 - 1.66) | 0.13 |
