# Supplementary material for: Transcriptomic recurrence score improves recurrence prediction for surgically treated patients with intermediate‐risk clear cell kidney cancer
Source: Cancer Med. 2022 Nov 17;12(5):6437–44. doi: 10.1002/cam4.5399 (PMC10028022; doi:10.1002/cam4.5399)
Supplement: Supplementary file 3 — Table S2 [file CAM4-12-6437-s002.docx]

**Table S2:** Univariable Fine and Gray model results for recurrence in SSIGN intermediate risk patients by eligibility for adjuvant therapy.

| **Adjuvant Eligible Patients** | | | |
| --- | --- | --- | --- |
| Variable | Subdist. Hazard Ratio (95% CI) | p-value | Patients (Events) |
| Recurrence Score Int-High vs Low | 2.43 (0.70 - 8.49) | 0.16 | 44 (9) |
| Recurrence Score Continuous | 2.37 (0.63 - 8.90) | 0.20 | 44 (9) |
| **Adjuvant Ineligible Patients** | | | |
| Variable | Subdist. Hazard Ratio (95% CI) | P-value | Patients (Events) |
| Recurrence Score Int-High vs Low | 2.15 (0.93 - 4.95) | 0.07 | 112 (22) |
| Recurrence Score Continuous | 3.38 (1.50 - 7.58) | 0.003 | 112 (22) |
